# Sugary drink consumption and risk of kidney and bladder cancer in Japanese adults

**DOI:** 10.1038/s41598-021-01103-x

**Published:** 2021-11-04

**Authors:** Chi Yan Leung, Sarah Krull Abe, Norie Sawada, Junko Ishihara, Ribeka Takachi, Taiki Yamaji, Motoki Iwasaki, Masahiro Hashizume, Manami Inoue, Shoichiro Tsugane

**Affiliations:** 1 Department of Global Health Policy, Graduate School of Medicine, The University of Tokyo, Tokyo, Japan; 2 Epidemiology and Prevention Group, Division of Prevention, Center for Public Health Sciences, National Cancer Center, 5-1-1 Tsukiji, Chuo-ku, Tokyo, 104-0045 Japan; 3 Department of Food and Life Science, Azabu University, Kanagawa, Japan; 4 Department of Food Science and Nutrition, Graduate School of Humanities and Sciences, Nara Women’s University, Nara, Japan; 5 Department of Cancer Epidemiology, Graduate School of Medicine, The University of Tokyo, Tokyo, Japan

**Keywords:** Risk factors, Public health, Epidemiology

## Abstract

Globally, sugary drinks are widely consumed, however, few epidemiologic studies have investigated the association between sugary drink consumption and risk of kidney and bladder cancer. We examined the association of sugary drinks with risk of kidney and bladder cancer in 73,024 participants from the Japan Public Health Center-based Prospective Study who reported no history of cancer. Sugary drink consumption was assessed using a validated food frequency questionnaire at study baseline (1995–1999). Individuals were followed to December 31, 2013. Multivariable Cox proportional hazards regression models were used to calculate hazard ratios (HR) and 95% confidence intervals (CIs). During 1,069,815 person years of follow-up, 169 kidney cancer and 297 bladder cancer cases were documented. After adjusting for potential confounders, no greater risk of kidney and bladder cancer was observed. However, sugary drink consumption was positively associated with the risk of kidney cancer (HR for 100 ml/day increase in consumption was 1.11 [95% CI 1.01–1.22]) and bladder cancer (HR for 100 ml/d increase in consumption was 1.11 [95% CI 1.01–1.22]) among women after exclusion of cases diagnosed in the first three years of follow-up. In this large prospective cohort, consumption of sugary drinks was significantly associated with a small increase in hazard ratio for kidney and bladder cancer among women after exclusion of cases diagnosed within the first three years.

## Introduction

Globally, the age-standardized incidence rates of kidney and bladder cancer have increased by 29% and 4% between 1990 and 2019, respectively^1^. In 2018, 403,262 new cases of kidney cancer and 549,393 new cases of bladder cancer were diagnosed^2^, accounting for the 16th- and 12th-most common cancers, respectively^2^. Despite these increasing trends, few modifiable lifestyle risk factors for kidney and bladder cancer have been identified, and no dietary factor has been clearly linked to risk^3,4^.

Sugary drinks, which are widely consumed globally, have been associated with overall and breast cancer incidence^5^ and have gained increasing interest in cancer development. The high fructose content of sugary drinks has been associated with postprandial hyperuricemia, which in turn contributes to a decline in renal function^6^. A study in more than one million participants aged 40 years or older showed a positive association between reduced kidney function (eGFR < 30) and risk of kidney cancer^7^. In addition, previous studies found that the consumption of sugary drinks was associated with diabetes, hypertension, and obesity^8^ which are known risk factors for kidney cancer^3,9^. Although there are several plausible mechanisms for how sugary drink consumption could lead to urinary bladder carcinogenesis^10,11^, their relative strengths have not been fully elucidated. On one hand, the urogenous hypothesis states that higher amounts of fluid intake may reduce bladder cancer risk by increasing micturition frequency and reducing the exposure time of the bladder to carcinogenic substances^10^, while on the other, contact of potential carcinogens in cola-type beverages with the bladder urothelium, such as 4-methylimidazole (IARC Group 2B carcinogen), may increase risk of bladder cancer^11^. In addition, sugary drinks have a high glycemic index, which was associated with bladder cancer risk in a meta-analysis of four studies (pooled odds ratio 1.25, 95% CI 1.11–1.41, *I*^2^ 0.0%)^12^.

To date, evidence regarding the association of sugary drink consumption with kidney and bladder cancer is sparse, and findings are inconsistent^13–16^. To our knowledge, no study has investigated potential mediators and modifiers of the albeit putative effect. In addition, there are concerns regarding reverse causality in prior literature, wherein early symptoms of undiagnosed cancer may affect sugary drink consumption habits. To fill this knowledge gap, we conducted a large prospective cohort study to investigate the association between sugary drink consumption and subsequent risk of kidney and bladder cancer over a 16-year median follow-up. We also examined reverse causation and potential effects of mediation and modification by the prespecified variables of body mass index (BMI), diabetes, and hypertension.

## Methods

### Study population

The Japan Public Health Center-based Prospective Study (JPHC Study) included 140,420 Japanese aged 40–69 years at the inception of the study, recruited from the general population in 1990 to 1994 in 11 prefectural public health center (PHC) areas. In 1990, 61,595 individuals were enrolled in Cohort I (Akita, Iwate, Nagano, Okinawa-Chubu, and Tokyo) and 78,825 were enrolled in 1993 in Cohort II (Ibaraki, Kochi, Niigata, Nagasaki, Osaka, and Okinawa-Miyako). At enrollment, participants answered self-administered food frequency questionnaires (FFQs) and provided information on their medical history and lifestyle. An expanded FFQ was sent at five-year follow-up to collect updated dietary information. For the current analysis, we used data from the five-year survey as baseline because it included a more comprehensive assessment of sugary beverage intake (eight sugary drink items) than that used at enrollment (two sugary drink items). Because of a lack of cancer incidence data, 7097 participants living in the Tokyo area were excluded. We further excluded non-Japanese nationals, (n = 51), pre-commencement migration (n = 188), those who had incorrect birth date (n = 7), duplicate registration (n = 10), or died or moved away from the study area before baseline of this analysis (n = 12,166). Additionally, we excluded those with cancer (n = 4,103), had not completed the follow-up questionnaires (n = 22,704), had the highest or lowest 2.5 percentile of calorie intake (< 993 or > 4204 kcal/day for men and < 837 or > 3683 kcal/d for women) (n = 5675), or had missing covariate information (n = 15,395). Finally, this resulted in the inclusion of 73,024 participants (33,094 men and 39,930 women). Table S1 presents key characteristics of included and excluded participants. Enrolled individuals were informed of the objectives of the study. Informed consent was obtained from all participants when completing the baseline questionnaire. The National Cancer Center Japan institutional review board has approved this study (approval number: 2001-021).

### Dietary assessment

The 5-year follow-up FFQ with 138 food and beverage items was validated using 14- or 28-day dietary records. Validation of the questionnaire has been published elsewhere^17,18^. In the present analysis, we defined sugary drinks as beverages that contain caloric sweeteners, which included 100% fruit juices (orange juice and apple juice), beta-carotene-fortified beverages, calcium-fortified beverages, canned coffee, carbonated beverages, lactic acid bacteria beverages, and vitamin-fortified beverages^19,20^. Lactic acid bacteria beverages are dairy products containing probiotics^21^. Sugar-sweetened tea and artificially sweetened beverages were not included in the FFQ. In the FFQ, nine options from “Never”, “1–2 times/week”, “3–4 times/week”, “5–6 times/week”, “1 cup/day”, “2–3 cups/day”, “4–6 cups/day”, “7–9 cups/day”, to “10 or more cups/day” were used to assess how often participants consumed sugary drinks. Standard portion size was defined as 250 ml for carbonated beverages and canned coffee; 200 ml for 100% fruit juices (orange juice and apple juice), beta-carotene fortified beverages, calcium-fortified beverages, and vitamin-fortified beverages; and 65 ml for lactic acid bacteria beverages. In the cohort, Spearman’s coefficients for validity were 0.32 (men) and 0.21 (women). Reproducibility of the FFQ was assessed through two FFQs one year apart, showing Spearman’s coefficients for reproducibility of 0.63 (men) and 0.56 (women)^19,20^.

### Case ascertainment

The occurrence of incident cancer was identified through record linkage to population cancer registries and by active patient notification from hospitals in the study areas, consisting of a supplement to death certificate files. Among kidney cancer cases, 6.9% were notified by death certificate (DCN), and 4.6% were ascertained by Death Certificate Only (DCO). For bladder cancer cases, DCN was 4.9% and DCO was 2.3%. The Third Edition of the International Classification of Disease for Oncology (ICD-10) was used to identify kidney cancer (C64) and bladder cancer (C67)^22^.

### Statistical analysis

Individuals were followed and person-years for each individual were calculated from the date of completion of the questionnaires in 1995 and 1998–1999 to the date of moving out of the study areas, death, or the study end date (31 December 2013; except Osaka, 31 December 2012), whichever occurred first. We categorized participants according to total daily sugary drink consumption (ml) rather than frequency owing to the different standard portion sizes for beverage items. Sugary drink consumers were divided into two categories based on sex-specific median consumption level (< 254 ml/day and ≥ 254 ml/day for men, and < 134 ml/day and ≥ 134 ml/day for women). The choice of median values was based on the consumption distributions of the present study, which allowed us to balance sample size between groups. Non-consumers were used as the reference category. Hazard ratios (HR) with 95% confidence intervals (CIs) over intake categories and by each 100-ml increase were evaluated using Cox proportional hazards regression models stratified by sex and adjusted for age (year, continuous); study area (10 PHC areas); history of diabetes (yes or no)^23–25^; body mass index (BMI) (< 18.5, 18.5– < 25, 25– < 30, or 30– < 45)^26^; hypertension history (yes or no)^27^; smoking status and intensity (never; former; current: < 20 cigarettes/day; current: ≥ 20 cigarettes/day)^28^; consumption of alcohol (ethanol gram/week, continuous)^29^, coffee (g/day)^30^, fruit (g/day, tercile), vegetables (g/day, tercile), Japanese-style confectionery (g/day, tercile), chocolate (g/day, tercile), biscuits (g/day, tercile), and cake (g/day, tercile); and physical activities (metabolic equivalent task, hours/day, tercile); height (cm, tercile); and total calorie intake (kcal/day, continuous). Figure S1 presents the directed acyclic graph used to determine the minimal adjustment set. The association of sugary drink consumption with risk of kidney and bladder cancer was also assessed across sex.

In sensitivity analysis, cancer cases occurring in the first three years of follow-up were excluded to minimize reverse causality. To estimate the degree to which the associations were mediated by obesity, diabetes, and hypertension, we repeated the analysis without adjustment for BMI, history of diabetes, and history of hypertension. In addition, subgroup analysis was performed to examine whether the associations between consumption of sugary drinks and risk of kidney and bladder cancer were modified by body mass index (kg/m^2^; < 25 or ≥ 25), history of diabetes (yes or no), and history of hypertension (yes or no). Likelihood ratio tests were used to test for interactions. We used Schoenfeld residuals to examine the proportionality of hazards for those covariates included in the Cox model, and no violation was found. In each model, a linear trend across categories was also tested using the median intake of each category as a continuous variable. To examine potential nonlinear associations, we conducted a likelihood ratio test in order to compare the model with linear and cubic spline terms for sugary drink consumption to the model only with linear term. Restricted cubic splines with five knots were used (placed at the 5th, 27.5th, 50th, 72.5th, and 95th percentiles)^31^. All statistical analyses were conducted using Stata STATA 14 (Stata Corp., College Station, TX, USA), with *P* < 0.05 considered statistically significant.

### Ethics approval and consent to participate

The study was conducted in compliance with the provisions of the Declaration of Helsinki. The study protocol was approved by the Institutional Review Board of the National Cancer Center, Japan. The participants were informed of the study objectives, and those who completed the survey questionnaire were regarded as consenting to participation.

## Results

**Figure 1:**
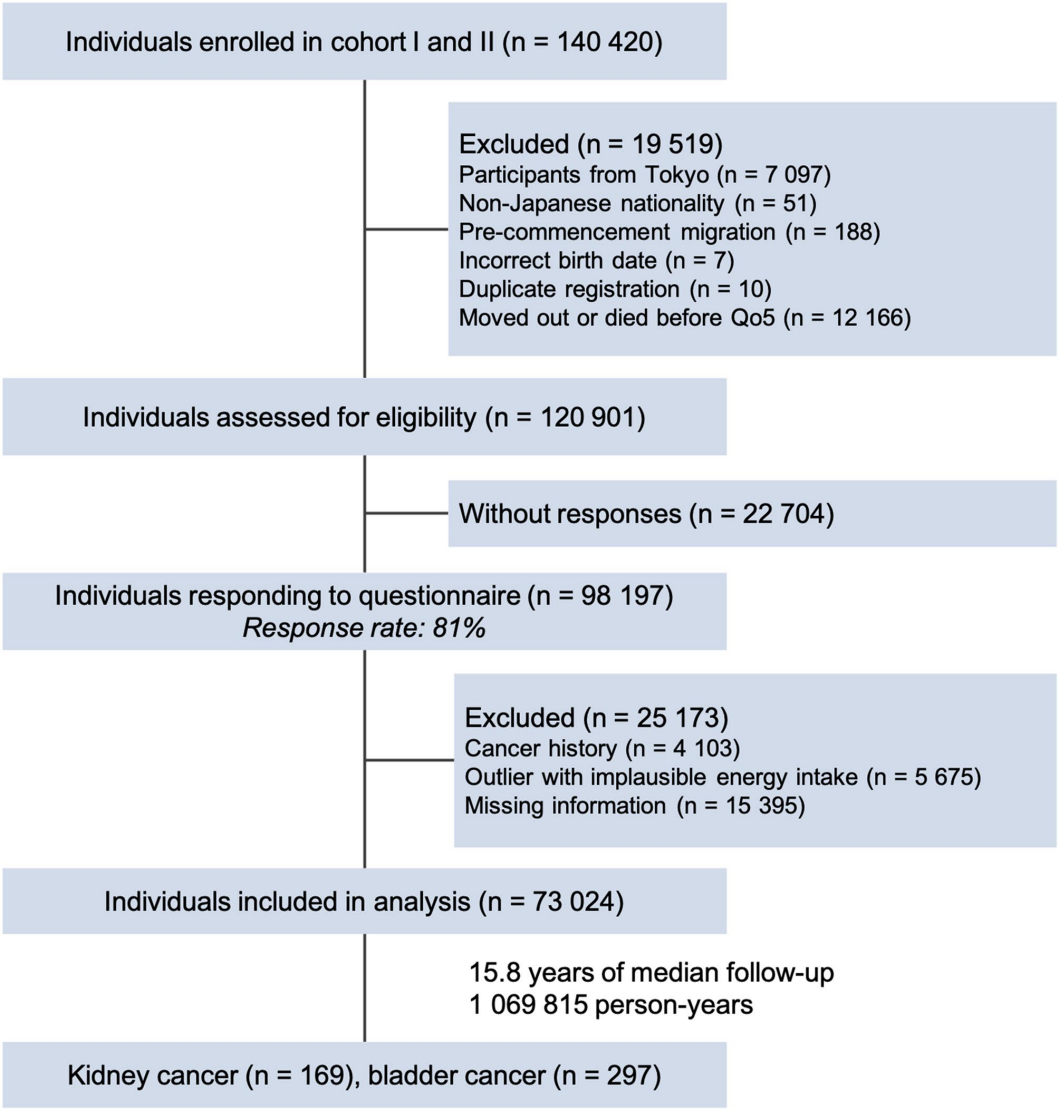
Participant flow chart.

During a median follow-up of 15.8 years (range 0 to 18.9 years; 1,069,815 person-years) among participants in the JPHC Study, 169 kidney and 297 bladder cancers were ascertained (Fig. 1). Participant baseline characteristics according to sugary drink consumption level are presented in Table 1. Compared with non-consumers, sugary drink consumers tended to be current smokers, younger, and with a higher consumption of total energy. In contrast, non-consumers were more likely to consume alcohol and have a past history of hypertension and diabetes. Women who consumed greater amounts of sugary drinks had a higher BMI than non-consumers.

**Table 1:** Baseline characteristic of participants according to sugary drink consumption in Japanese men and women.

Characteristic	Category of sugary drink consumption		
**Men**			
Intake category, ml/day	Non-consumers	> 0–254	≥ 254
Number	4363	14,366	14,365
Age, mean (SD), year	58.2 (7.9)	57.0 (7.6)	55.2 (7.6)
Physical activity, mean (SD), METS-h/day	31.7 (6.4)	32.7 (6.8)	33.3 (6.9)
BMI, mean (SD), kg/m^2^	23.6 (2.9)	23.7 (2.8)	23.6 (2.9)
Past history of hypertension, %	18.4	15.9	12.4
Past history of diabetes, %	14.9	9.7	6.4
Current smoker, %	42.4	43.4	52.8
Current alcohol consumption, %	75.5	75.6	68.7
Total energy, mean (SD), kcal/day	1958.4 (567.8)	2204.7 (628.4)	2222.9 (644.3)
Vegetables, mean (SD), g/day	139.0 (92.1)	133.9 (85.8)	125.3 (76.3)
Fruit, mean (SD), g/day	70.3 (70.1)	83.5 (68.7)	101.2 (78.6)
Coffee consumption, mean (SD), g/day	127.5 (196.1)	121.7 (188.3)	128.8 (181.8)
**Women**			
Intake category, ml/day	Non-consumers	> 0–134	≥ 134
Number	6380	16,775	16,775
Age, mean (SD), year	57.0 (8.0)	56.8 (7.7)	56.2 (7.7)
Physical activity, mean (SD), METS-h/day	31.6 (5.6)	32.1 (5.7)	32.3 (5.9)
BMI, mean (SD), kg/m^2^	23.3 (3.2)	23.5 (3.1)	23.6 (3.2)
Past history of hypertension, %	13.4	13.5	13.2
Past history of diabetes, %	7.2	4.1	2.8
Current smoker, %	5.4	4.7	6.4
Current alcohol consumption, %	19.5	18.1	17.9
Total energy, mean (SD), kcal/day	1,660.7 (483.2)	1,915.6 (548.4)	1,928.7 (567.7)
Vegetables, mean (SD), g/day	237.1 (139.6)	226.9 (125.1)	214.4 (116.1)
Fruit, mean (SD), g/day	197.1 (161.3)	222.1 (152.8)	261.0 (163.9)
Coffee consumption, mean (SD), g/day	107.1 (143.1)	99.4 (138.1)	97.8 (131.4)

*SD* standard deviation, *BMI* body mass index, *kg* kilogram, *m* meter, *kcal* kilocalorie, *g* gram. Sugary drinks included beta-carotene-fortified beverages, calcium-fortified beverages, canned coffee, carbonated beverages, 100% fruit juices (apple juice and orange juice), lactic acid bacteria beverages, and vitamin-fortified beverages.

In minimally adjusted and multivariable analyses, there was no association of sugary drink consumption with risk of kidney cancer in men and women combined (Table 2). A similar lack of association was observed when we analyzed men and women separately. Compared with non-consumers, men with a consumption of ≥ 254 ml per day of sugary drinks had a HR of 0.89 (95% CI 0.49–1.62, *P* = 0.71) for kidney cancer. In women, the multivariable HR for kidney cancer among those who had consumed ≥ 134 ml per day was 0.93 (95% CI 0.43–2.03, *P* = 0.86). Further, no association was found between sugary drink consumption and bladder cancer among both women and men (Table 2). Compared to non-consumers, the multivariable-adjusted HR for bladder cancer among men was 0.73 (95% CI 0.49–1.08, *P* = 0.11) for those who consumed ≥ 254 ml per day. Consumption was not associated with increased bladder cancer risk among women (non-consumers vs. ≥ 134 ml per day; HR, 0.95; 95% CI 0.45–2.03, *P* = 0.90). In mediation analysis, the results remained unchanged after exclusion of BMI and histories of diabetes and hypertension from the model.

**Table 2:** Hazards for kidney and bladder cancer incidence according to intake of sugary drinks in Japanese men and women.

	Category of sugary drink consumption, HR (95% CI)			*P* value for trend	Per 100 ml per day incremental, HR (95% CI)
**Kidney cancer**					
Both					
Intake category, ml/day	Non-consumers	Men: > 0–254 Female: > 0–134	Men: ≥ 254 Female: ≥ 134		
No. of cases	27	71	71		
Person-years	152,902	454,565	462,348		
Minimally adjusted^a^	1 [reference]	0.86 (0.55–1.34)	0.88 (0.56–1.38)	0.80	1.02 (0.96–1.08)
Multivariable model^b^	1 [reference]	0.88 (0.56–1.38)	0.92 (0.57–1.47)	0.93	1.02 (0.96–1.08)
Excluding 3 years^b^	1 [reference]	0.90 (0.54–1.50)	1.08 (0.64–1.81)	0.56	1.04 (0.99–1.10)
Without HTN, DM, BMI^c^	1 [reference]	0.88 (0.56–1.38)	0.92 (0.57–1.46)	0.92	1.02 (0.96–1.08)
Men					
Intake category, ml/day	Non-consumers	> 0–254	≥ 254		
No. of cases	17	49	47		
Person-years	58,605	199,955	206,381		
Minimally adjusted^a^	1 [reference]	0.89 (0.51–1.55)	0.90 (0.51–1.59)	0.86	1.01 (0.95–1.08)
Multivariable model^b^	1 [reference]	0.89 (0.50–1.56)	0.89 (0.49–1.62)	0.84	1.01 (0.95–1.08)
Excluding 3 years^b^	1 [reference]	0.82 (0.44–1.50)	0.94 (0.50–1.76)	0.78	1.03 (0.96–1.09)
Without HTN, DM, BMI^c^	1 [reference]	0.90 (0.51–1.58)	0.91 (0.50–1.64)	0.87	1.01 (0.95–1.08)
Women					
Intake category, ml/day	Non-consumers	> 0–134	≥ 134		
No. of cases	10	22	24		
Person-years	94,297	254,610	255,967		
Minimally adjusted^a^	1 [reference]	0.78 (0.37–1.67)	0.80 (0.37–1.70)	0.74	1.04 (0.91–1.19)
Multivariable model^b^	1 [reference]	0.83 (0.38–1.79)	0.93 (0.43–2.03)	0.91	1.07 (0.95–1.21)
Excluding 3 years^b^	1 [reference]	1.06 (0.41–2.72)	1.37 (0.54–3.50)	0.37	1.11 (1.01–1.22)
Without HTN, DM, BMI^c^	1 [reference]	0.81 (0.38–1.75)	0.91 (0.42–1.98)	0.95	1.07 (0.95–1.20)
**Bladder cancer**					
Both					
Intake category, ml/day	Non-consumers	Men: > 0–254 Female: > 0–134	Men: ≥ 254 Female: ≥ 134		
No. of cases	50	126	121		
Person-years	152,902	454,565	462,348		
Minimally adjusted^a^	1 [reference]	0.84 (0.60–1.17)	0.85 (0.61–1.20)	0.57	1.02 (0.98–1.06)
Multivariable model^b^	1 [reference]	0.81 (0.58–1.13)	0.79 (0.56–1.12)	0.33	1.01 (0.97–1.06)
Excluding 3 years^b^	1 [reference]	0.80 (0.56–1.14)	0.79 (0.54–1.14)	0.33	1.01 (0.96–1.06)
Without HTN, DM, BMI^c^	1 [reference]	0.80 (0.57–1.12)	0.78 (0.55–1.10)	0.29	1.01 (0.97–1.06)
Men					
Intake category, ml/day	Non-consumers	> 0–254	≥ 254		
No. of cases	40	96	93		
Person-years	58,605	199,955	206,381		
Minimally adjusted^a^	1 [reference]	0.74 (0.51–1.07)	0.75 (0.51–1.09)	0.37	1.01 (0.96–1.05)
Multivariable model^b^	1 [reference]	0.73 (0.50–1.06)	0.73 (0.49–1.08)	0.34	1.01 (0.96–1.05)
Excluding 3 years^b^	1 [reference]	0.74 (0.50–1.11)	0.71 (0.47–1.09)	0.29	0.99 (0.94–1.05)
Without HTN, DM, BMI^c^	1 [reference]	0.72 (0.49–1.05)	0.71 (0.48–1.06)	0.31	1.00 (0.96–1.05)
Women					
Intake category, ml/day	Non-consumers	> 0–134	≥ 134		
No. of cases	10	30	28		
Person-years	94,297	254,610	255,967		
Minimally adjusted^a^	1 [reference]	1.25 (0.61–2.58)	1.28 (0.61–2.67)	0.64	1.10 (1.01–1.20)
Multivariable model^b^	1 [reference]	1.10 (0.53–2.28)	0.95 (0.45–2.03)	0.70	1.08 (0.97–1.20)
Excluding 3 years^b^	1 [reference]	0.96 (0.44–2.10)	1.01 (0.46–2.23)	0.89	1.11 (1.01–1.22)
Without HTN, DM, BMI^c^	1 [reference]	1.11 (0.53–2.31)	0.97 (0.46–2.06)	0.74	1.08 (0.97–1.20)

*HR* hazard ratio, *CI* confidence interval, *No.* number.

^a^Minimally adjusted model adjusted for age (year); public health center (10 areas); smoking status and intensity (never; former; current: < 20 cigarettes per day; current: ≥ 20 cigarettes per day); physical activities (metabolic equivalent task, hours per day, tercile); and intake of total energy (kcal per day) (continuous).

^b^Multivariable Cox regression model adjusted for age (year); public health center (10 areas); body mass index (< 18.5, 18.5 to < 25, 25 to < 30, or 30 to < 45); history of hypertension (yes or no); history of diabetes (yes or no), smoking status and intensity (never; former; current: < 20 cigarettes per day; current: ≥ 20 cigarettes per day); consumption of alcohol (ethanol gram per week), coffee (gram per day), fruit (g per day, tercile), vegetables (g per day, tercile), biscuits (g per day, tercile), cake (g per day, tercile), chocolate (g per day, tercile), and Japanese-style confectionery (g per day, tercile); physical activities (metabolic equivalent task, hours per day, tercile); height (cm, tercile); and intake of total energy (kcal per day) (all continuous).

^c^History of hypertension, history of diabetes, and body mass index were not adjusted for. Sugary drinks included beta-carotene-fortified beverages, calcium-fortified beverages, canned coffee, carbonated beverages, 100% fruit juices (apple juice and orange juice), lactic acid bacteria beverages, and vitamin-fortified beverages.

To minimize reverse causation, we excluded 23 kidney cancer and 32 urinary bladder cancer cases that were diagnosed within the first three years of follow-up. In categorical analysis, no association with kidney and bladder cancer was found. In continuous analyses, consumption of sugary drinks was marginally positively associated with the risk of kidney cancer (HR for 100 ml per day increase, 1.11; 95% CI 1.01–1.22, *P* = 0.03) and bladder cancer (HR for 100 ml per day increase, 1.11; 95% CI 1.01–1.22, *P* = 0.03) among women (Table 2). Restricted cubic spline regression analyses showed that the relations between sugary drink consumption and risk of kidney (men, *P* = 0.13; women, *P* = 0.18) and bladder cancer (men, *P* = 0.67; women, *P* = 0.06) were consistent with linear associations. No differences were observed in the associations of sugary drink consumption with risk of kidney and bladder cancer when stratified by BMI and histories of diabetes and hypertension (Table 3).

**Table 3:** Hazards for kidney and bladder cancer incidence according to intake of sugary drinks, stratified by body mass index, history of hypertension and diabetes.

Intake category, ml/day	Category of sugary drink consumption, HR (95% CI)			*P* for interaction
	Non-consumers	Men: > 0–254 Female: > 0–134	Men: ≥ 254 Female: ≥ 134	
**Both sexes**				
Kidney cancer				
BMI^a^				0.42
BMI < 25	1 [reference]	0.99 (0.56–1.74)	0.90 (0.50–1.64)	
BMI ≥ 25	1 [reference]	0.66 (0.31–1.44)	0.89 (0.41–1.93)	
History of hypertension ^b^				0.92
No	1 [reference]	0.82 (0.49–1.37)	0.90 (0.53–1.53)	
Yes	1 [reference]	1.04 (0.39–2.75)	0.88 (0.31–2.50)	
History of diabetes^c^				0.79
No	1 [reference]	0.96 (0.58–1.56)	0.98 (0.59–1.63)	
Yes	1 [reference]	0.50 (0.13–1.87)	0.54 (0.12–2.37)	
Bladder cancer				
BMI^a^				0.76
BMI < 25	1 [reference]	0.77 (0.51–1.15)	0.77 (0.51–1.17)	
BMI ≥ 25	1 [reference]	0.89 (0.48–1.65)	0.80 (0.42–1.52)	
History of hypertension^b^				0.88
No	1 [reference]	0.80 (0.55–1.16)	0.78 (0.53–1.15)	
Yes	1 [reference]	0.86 (0.40–1.84)	0.83 (0.37–1.87)	
History of diabetes^c^				0.70
No	1 [reference]	0.78 (0.55–1.12)	0.74 (0.51–1.07)	
Yes	1 [reference]	0.86 (0.32–2.27)	1.29 (0.46–3.64)	

*HR* hazard ratio, *CI* confidence interval, *No.* number. Multivariable Cox regression model adjusted for age (year); public health center (10 areas); body mass index (< 18.5, 18.5 to < 25, 25 to < 30, or 30 to < 45); history of hypertension (yes or no); history of diabetes (yes or no), smoking status and intensity (never; former; current: < 20 cigarettes per day; current: ≥ 20 cigarettes per day); consumption of alcohol (ethanol gram per week), coffee (gram per day), fruit (g per day, tercile), vegetables (g per day, tercile), biscuits (g per day, tercile), cake (g per day, tercile), chocolate (g per day, tercile), and Japanese-style confectionery (g per day, tercile); physical activities (metabolic equivalent task, hours per day, tercile); height (cm, tercile); and intake of total energy (kcal per day) (all continuous).

^a^Body mass index was not adjusted.

^b^History of hypertension was not adjusted.

^c^History of diabetes was not adjusted. Sugary drinks included beta-carotene-fortified beverages, calcium-fortified beverages, canned coffee, carbonated beverages, 100% fruit juices (apple juice and orange juice), lactic acid bacteria beverages, and vitamin-fortified beverages.

## Discussion

In this large population-based prospective cohort of 73,024 participants with a 15.8-year median follow-up, we found null associations between sugary drink consumption and risk of kidney and bladder cancers. However, our results suggest that higher sugary drink consumption was associated with a greater risk of kidney and bladder cancers among women after excluding kidney and bladder cancer cases diagnosed within three years after baseline assessment.

Sugary drink consumption has been hypothesized to increase kidney cancer risk^7,32–39^, but two previous studies did not find positive associations^13,14^. In a pooled analysis involving 13 prospective studies, no association was observed between soda consumption and renal cell cancer (pooled multivariable relative risk increment of 355 ml per day = 1.02; 95% CI 0.9 to 1.06)^14^. Consistently, higher consumption of sugar-sweetened soft drinks was not associated with kidney cancer risk in the Melbourne Collaborative Cohort Study (≥ 1 cup/day vs < 1 cup/month; HR 1.32; 95% CI 0.79 to 2.19)^13^. In the current study, the null association of sugary drinks with risk of kidney cancer in the primary analysis accords with results from previous studies^13,14^. However, sugary drink consumption at baseline may have been changed or under-reported for participants with obesity, hypertension, or diabetes, which may bias the associations downward. To address this, we adjusted these covariates in our multivariable models and assessed the association after exclusion of participants diagnosed with cancer during the first three years. After exclusion, positive associations between sugary drink consumption and risk of kidney and bladder cancer were observed in women. Of note, this exclusion may have reduced the statistical power of our analyses. Reverse causation was also assessed in the Melbourne Collaborative Cohort Study, but the results remained nonsignificant after excluding cases diagnosed in the first 2 years. The lack of significance in the Melbourne study might have been due to the limited number of cases (146 kidney cancer cases before exclusion). Nevertheless, the potential influence of changes in diet and lifestyle in the present study cannot be eliminated completely, and thus risk may be underestimated.

Two previous studies on the association of sugary drink consumption with bladder cancer have been conducted, albeit with conflicting results. In the European Prospective Investigation into Cancer and Nutrition (EPIC), increased consumption of soft drinks was weakly associated with an increased risk of urothelial cell carcinoma (HR for 100 ml incremental, 1.06; 95% CI 1.01 to 1.12)^15^. On the other hand, the Health Professionals Follow-up Study (HPFS) reported a null association of bladder cancer risk with consumption of soda and lemonade (HR for 240 ml incremental, 0.99; 95% CI 0.90 to 1.08)^16^. Reverse causation was not assessed in these two studies. Our findings in the primary analysis did not support the association of sugary drinks with bladder cancer risk. The length of time that carcinogens are in contact with urothelial cells of the bladder has been postulated to be associated with bladder cancer risk and may explain the null association^10^. Higher fluid consumption increases urination volume and frequency, thereby reducing contact time and likely counterbalancing the potential carcinogenic effects of sugary drinks on bladder^10^. However, similar to kidney cancer, a positive association was observed in women after the exclusion of cancer cases diagnosed within the first three years of follow up. Therefore, the real associations in the primary analyses may be biased downward to null due to reverse causality.

This large population-based prospective study, to our knowledge, was the first attempt to assess the association of sugary drink consumption with risk of kidney and bladder cancer in the Western Pacific region, where the percentage increases in the incidence of kidney (117.1%, 95% UI 88.2–152.2%) and bladder cancer (35%, 95% UI 17–59%) were the highest and second highest, respectively, among World Health Organization regions between 1990 and 2019^40^. The major strength of this study is that the comprehensive lifestyle and dietary information were collected prospectively, with a high follow-up rate and long follow-up duration; this reduced the potential for recall bias and selection bias. Our study also has several limitations that warrant discussion. Owing to the self-reported nature of the questionnaire, misclassification from measurement error is inevitable. Given the prospective design of the study, however, such errors are likely to be non-differential. Nevertheless, we acknowledge that the correlation coefficients for validity were low in the cohort and that the use of fixed sugary drink items in the questionnaire might have resulted in imprecise measurement. As with any observational study, a possible impact of residual confounding could not be totally ruled out. Although we adjusted for major confounders that may have impacted the risk of kidney and bladder cancer, residual confounding from unmeasured variables, occupational, or socioeconomic factors is possible.

## Conclusions

In summary, this large prospective cohort study demonstrated that sugary drink consumption was associated with slightly greater risks of kidney and bladder cancer in women, but not in men, after excluding incident cancers diagnosed in the first 3 years of follow-up. Additional studies with adequate cancer cases are needed to further examine the potential for reverse causation in this association.

## Supplementary Information

Supplementary Figure S1.

Supplementary Table S1.

## Data Availability

For information on how to apply for access to the JPHC data and/or biospecimens, please follow the instructions at http://epi.ncc.go.jp/en/jphc/805/8155.html.
