# Supplementary figures and images for: Sugary drink consumption and risk of kidney and bladder cancer in Japanese adults
Source: Sci Rep. 2021 Nov 4;11:21701. doi: 10.1038/s41598-021-01103-x (PMC8568905; doi:10.1038/s41598-021-01103-x)

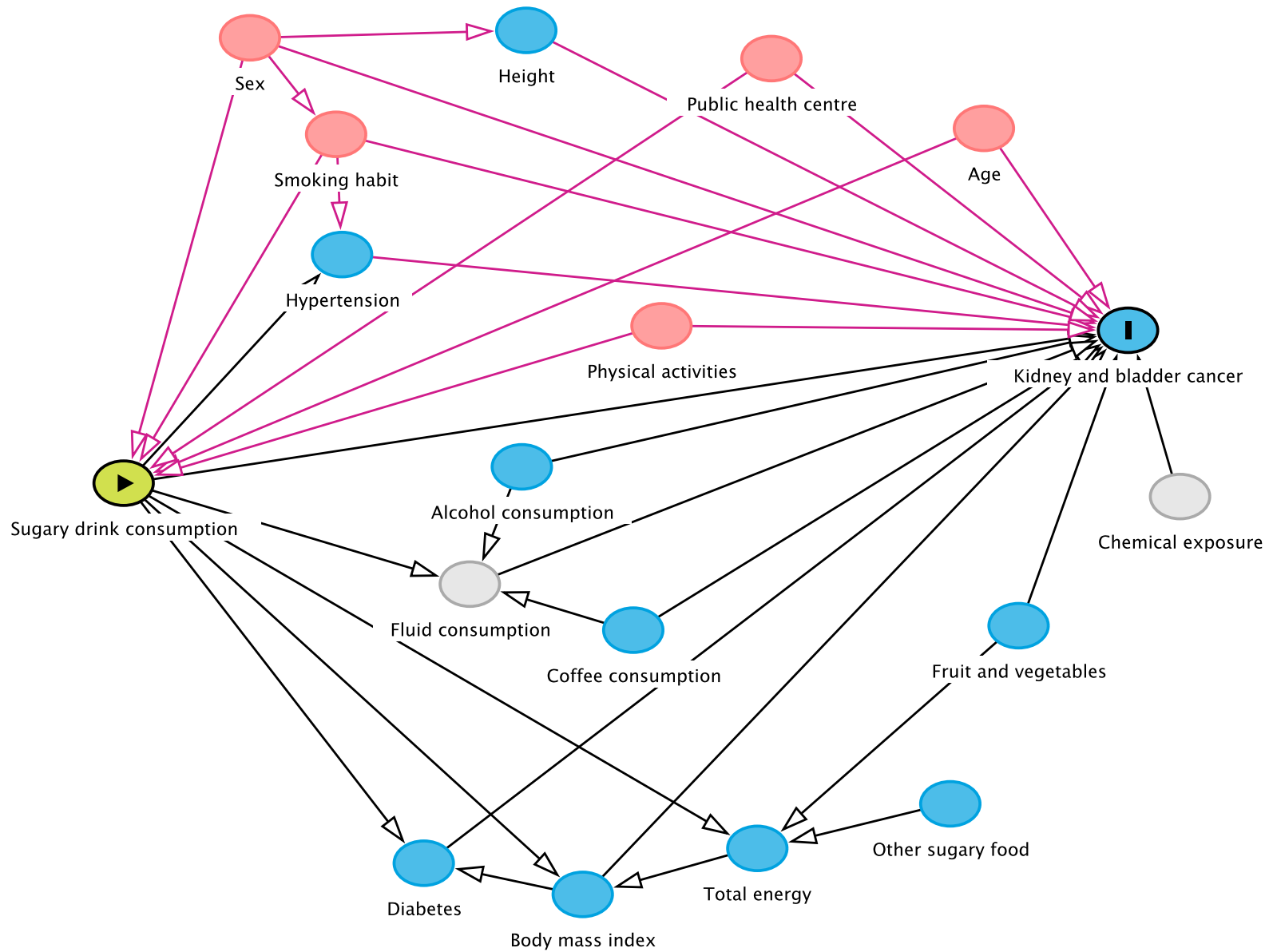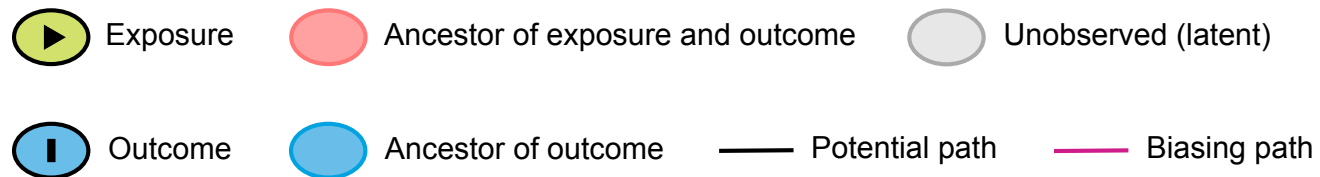

Supplement: Supplementary file 1 — Supplementary Figure S1. [file 41598_2021_1103_MOESM1_ESM.pdf]
