# Supplementary material for: Sugary drink consumption and risk of kidney and bladder cancer in Japanese adults
Source: Sci Rep. 2021 Nov 4;11:21701. doi: 10.1038/s41598-021-01103-x (PMC8568905; doi:10.1038/s41598-021-01103-x)
Supplement: Supplementary file 2 — Supplementary Table S1. [file 41598_2021_1103_MOESM2_ESM.docx]

**Table S1.** Key characteristics of included and excluded participants

| **Characteristic** | **Included participant**  **(n = 73,024)** | **Excluded participant**  **(n = 15,395)** | ***P*-value** |
| --- | --- | --- | --- |
| Men, % | 45.3% | 53.6% | 0.000 |
| Age, mean (SD), year | 56.5 (7.7) | 59.7 (8.2) | 0.000 |
| BMI, mean (SD), kg/m^2^ | 23.5 (3.0) | 23.5 (3.1) | 0.236 |
| Physical activity, mean (SD), METS-hour/day | 32.4 (6.3) | 31.8 (6.5) | 0.000 |
| Past history of diabetes, % | 6.3% | 6.8% | 0.033 |
| Past history of hypertension, % | 14.0% | 17.5% | 0.000 |
| Current smoker, % | 24.5% | 34.4% | 0.000 |
| Current alcohol consumption, % | 42.9% | 62.4% | 0.000 |
| Dietary intake ^a^ |  |  |  |
| Total energy, mean (SD), kcal/day | 2016.3 (610.6) | 1924.9 (655.7) | 0.000 |
| Vegetables, mean (SD), g/day | 181.4 (116.9) | 170.4 (128.0) | 0.000 |
| Fruit, mean (SD), g/day | 168.7 (147.7) | 157.9 (163.4) | 0.000 |
| Coffee consumption, mean (SD), g/day | 111.6 (161.5) | 98.4 (155.0) | 0.000 |

Abbreviations: n, number; SD, standard deviation; BMI, body mass index; kg, kilogram; m, meter; MET, metabolic equivalent of task; kcal, kilocalorie; g, gram. ^a^ All dietary intakes were energy-adjusted by the residual method.
